# Supplementary material for: Prospective Clinical Evaluation of the Diagnostic Accuracy of a Highly Sensitive Rapid Antigen Test Using Silver Amplification Technology for Emerging SARS-CoV-2 Variants
Source: Biomedicines. 2022 Nov 3;10(11):2801. doi: 10.3390/biomedicines10112801 (PMC9687987; doi:10.3390/biomedicines10112801)
Supplement: Supplementary file 1 [file biomedicines-10-02801-s001.zip › biomedicines-1915799-supplementary.pdf]

Supplementary Materials

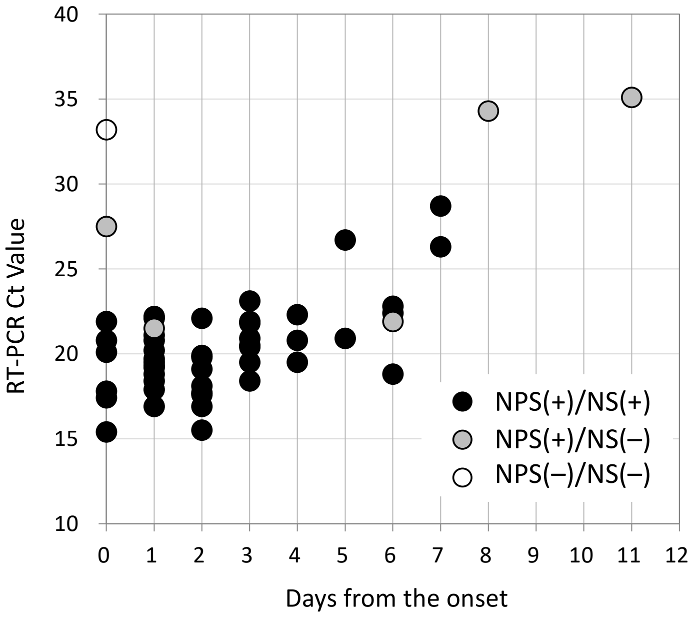

Figure S1. Relationship between Ct values and days after onset for 52 individuals and their antigen rapid diagnostic tests (Ag-RDTs).
